# Time Does Not Help Orangutans *Pongo abelii* Solve Physical Problems

**DOI:** 10.3389/fpsyg.2017.00161

**Published:** 2017-02-07

**Authors:** Johan Lind, Sofie Lönnberg, Tomas Persson, Magnus Enquist

**Affiliations:** ^1^Centre for the Study of Cultural Evolution, Stockholm UniversityStockholm, Sweden; ^2^Department of Zoology, Stockholm UniversityStockholm, Sweden; ^3^Lund University Cognitive Science, Lund UniversityLund, Sweden

**Keywords:** animal cognition, methodology, intelligence, orangutans, reasoning, thinking

## Abstract

Many questions in animal intelligence and cognition research are challenging. One challenge is to identify mechanisms underlying reasoning in experiments. Here, we provide a way to design such tests in non-human animals. We know from research in skill acquisition in humans that reasoning and thinking can take time because some problems are processed in multiple steps before a solution is reached (e.g., during mental arithmetics). If animals are able to learn through similar processes their decision making can be time consuming, and most importantly improve if more time to process information is allowed. We tested if performance of two Sumatran orangutans (*Pongo abelii*) increased in a two-choice experiment when they were allowed extra time before making their decisions, compared to when they were forced to decide immediately. We found that the performance of the orangutans did not depend on the time they were allowed to process the information before making their decisions. This methodology provides a potential avenue for empirical tests of mechanisms underlying reasoning in non-human animals.

## Introduction

A fundamental question in animal intelligence and cognition research is if, or to what extent, non-human animals have capacities for causal reasoning during learning and decision making (defined in, e.g., Emery and Clayton, 2004, see also Reznikova, 2010). It has been suggested, for example, that birds and apes can reason when learning physical and social tasks (e.g., Hill et al., 2011; Jelbert et al., 2015; Smirnova et al., 2015, but see Ghirlanda and Lind, 2017). However, there is no consensus regarding the extent of non-human animal reasoning capacities and many debates stem from disputes regarding methodological issues, for example with respect to insight learning (see discussion between, e.g., Bird and Emery, 2009 and Lind et al., 2009), cognitive maps (Tolman and Honzik, 1930; Anthony, 1959; Ciancia, 1991), and social learning (see e.g., Heyes, 2012 commenting on Horner et al., 2011).

That it is difficult to identify reasoning in non-human animals is well known (e.g., Anthony, 1959; Penn and Povinelli, 2007; Lind et al., 2009; Reznikova, 2010) and researchers have called for novel methodologies to improve identification of mental capacities (e.g., Povinelli and Vonk, 2003; Heyes, 2012). One problem has been to devise methods that can operationalize ideas about how animals perform mental operations, that is capturing what is meant by causal reasoning (Emery and Clayton, 2004), reasoning about mental states of others (Povinelli and Vonk, 2003), or if animals solve problems through insight (Bird and Emery, 2009; but see Lind et al., 2009). With inspiration from computer programming and human thinking, we here describe a way to identify reasoning capacities by examining if the time allowed to attend to a problem increases the likelihood of successfully solving that problem, and we provide data from an experiment with Sumatran orangutans (*Pongo abelii*). In addition, our test can control for operant conditioning, by some considered a prerequisite for testing cognition beyond associative learning (e.g., Pearce, 2008; Lind et al., 2009; Heyes, 2012; Neves Filho et al., 2015).

We find no reasons within learning theory (see e.g., Rescorla and Wagner, 1972; Pearce and Bouton, 2001) that decision making based on associative learning should improve if more time is allowed before a choice is made. And, we see important similarities between standard associative learning and what in computer programming is called early binding (Enquist et al., 2016). In early binding solutions to previously experienced problems are stored in a table. Accordingly, an animal that encounters a situation only has access to already established behaviors that, so to speak, can be picked from a table of suitable responses. In late binding, however, a decision-making mechanism is free to use detailed information in different ways. An animal capable of late binding can recall previous experiences, filter out useful ones, and reorganize information to figure out the solution to the problem at hand. Importantly, no ready-made, previously established, solutions must be stored and used. The way behaviors are described as flexible, insightful and caused by reasoning fit well within the concept of late binding (e.g., Tomasello and Call, 1997; Emery and Clayton, 2004; Tecwyn et al., 2012; Jelbert et al., 2014; Smirnova et al., 2015).

There is a large body of literature concerned with the relationship between behavior and time. Studies have measured, for instance, how long time it took a macaque to respond to visual discriminations of different difficulties (Sayers et al., 2015), that response time can vary with changing conditions in visual discrimination task (Beran et al., 2004) and whether time to make decisions regarding visual stimuli and video clips of hand movements correlated with a measurement of confidence in the decision (Patel et al., 2012). In our study, we did not measure response time but instead set time under experimental control by using two fixed durations so that decisions made by orangutans were forced to either occur immediately or after a set time delay.

Human reasoning, when comparable to late binding in computer programming, can take a long time. Let us illustrate. In humans, when calculating 3^∗^3 you are likely to give the correct answer instantaneously, it is something you just know as a result of extensive previous learning. However, if you are asked to calculate something less familiar, such as 13^∗^17, it might take a longer time because you have to perform an actual arithmetic operation. Now information must be reorganized in several steps to produce the correct answer (see e.g., Anderson, 1982; VanLehn, 1996). Problems can even take hours or days to solve. In dual process theory researchers often call this an explicit process (e.g., Sun, 2001), and Kahneman (2011) made this concept famous as slow thinking, characterized by slow and attention demanding mental processing. That higher performance is achieved in decision experiments when more time is allowed has been shown in children. Small children could only perform as well as older children if they were allowed 15 s extra time to think about the problem at hand (Pezdek and Miceli, 1982). The task was based on verbal and visual stimuli, presented in sequences, and subjects were required to distinguish between semantically relevant and irrelevant items. Other experiments have shown that human mental operations can take time. Here, humans and pigeons were exposed to a set of mental rotation tests and only humans needed more time to correctly match test stimuli to sample stimuli (Hollard and Delius, 1982). Nevertheless, it should be noted that additional time can only be beneficial if the problem to be solved is within the capacitiy of the test subject.

If more time for decision making improves problem solving, the process can depend on some kind of reasoning ability that includes reorganization of information. This means that an animal with a capacity for late binding should, if allowed enough time to process the information, solve problems with higher precision than animals without such a capacity. We tested this hypothesis with two Sumatran orangutans who were subjected to a set of two-choice problems in two treatments; they either had to make their choice immediately upon presentation, or they were given time before making their choice, thus allowing time for potential reorganization of information.

## Materials and Methods

### Ethics Statement

The study was approved by the Uppsala regional ethics committee (No. C169/10), and Lund University Primate Research Station at Furuvik Zoo is an approved cognitive research facility (No. 31-2599/09). Experiments were performed in a compartment that is part of the everyday facilities. Only one animal at a time was allowed in the experimental compartment to make sure that all tests were novel at the time of testing for both animals. If an orangutan did not want to proceed with an experiment it was always allowed to leave the experimental compartment. However, this did not happen. All applicable international, national, and institutional guidelines for the care and use of animals were followed. All procedures performed here were in accordance with the ethical standards of the institution and practice at which the studies were conducted.

### Procedure

Both orangutans were experienced test subjects, the female Igelchen born in 1985, and the male Naong born in 1990 (e.g., Osvath and Osvath, 2008; Osvath and Persson, 2013). They were capable of manually indicating objects in choice situations. They were also capable of all actions needed in the experiment, such as raking, pulling strings, drinking from flasks using straws, and pour objects (such as peanuts) from containers.

**FIGURE 1:**
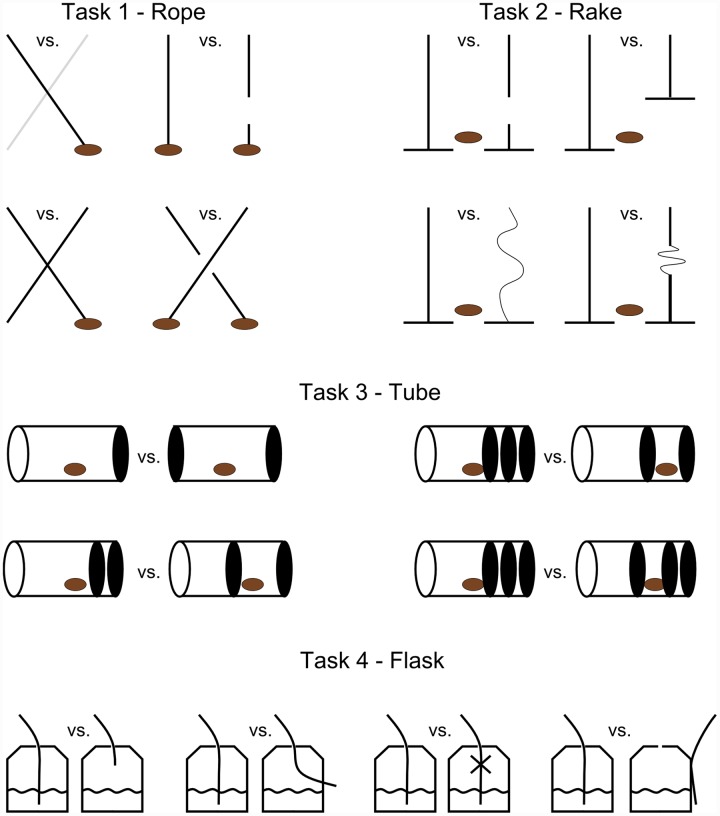
**Illustration of experimental tasks**. In task 1 only one of the ropes could result in a peanut being pulled in. Where ropes were crossed we used both differently colored ropes (light and dark) and ropes of the same color. In task 2, only one of two rakes was functional. The zig-zag forms represent thin and slack ropes which could not be used to push the rakes forward. Therefore, the rakes were too short for a peanut to be raked in. In task 3 the orangutans were not allowed to manipulate any object but instead choose the tube where a peanut could fall out without being blocked by a styrofoam wall (solid black). In task 4 the orangutans were allowed to choose between transparent flasks where one flask in each session had a straw in a functional position, whereas the other four were too short, not in contact with the fluid, was blocked to prevent flow of fluid, and outside the flask, respectively. Note that due to unclear results from the preference test in task 4 we did not use data from this task in our analysis.

We used a two-choice method and presented, out of reach, two similar objects on a tray 30 cm apart. Only one of the two objects was functional and could result in a reward (a peanut or fruit drink). The other object was non-functional in a visually overt way, such as a broken string, a non-functional slack rake, a tube with walls that prevented peanuts from falling out, or a straw that did not allow the orangutan to drink the fluid (**Figure 1**). A trial started by the removal of an opaque cardboard screen behind which stimuli had been hidden. When subjects indicated their choice the non-chosen object was immediately taken away, and the tray was pushed toward the orangutan so it could reach the indicated object and use it to get the reward. Reaching the reward was only possible if a functional tool was chosen. In the tube task (all tasks are presented below), however, the orangutans were not allowed to manipulate any objects. Instead, when the non-chosen object was taken away, the experimenter turned the chosen tube around so that the reward could fall down on the tray, if the functional tube was chosen. Alternatively, if the non-functional tube was chosen the food reward remained trapped in the tube (**Figure 1**).

We tested four versions of four different tasks (see below). Thus each subject was tested on 16 problems. Each problem was tested within one session with six repetitions (inter-trial intervals at approximately 60 s). Both orangutans were thus exposed to 16 different problems, with 6 trials for each problem. The setup was pseudo-randomized so that the attainable rewards were balanced across left and right sides. Both orangutans did the four tasks in the following order: rope, rake, tube, flask.

To test for time-dependent decision making we introduced a time delay in half of all trials. These trials were identical apart from that now the orangutans had the opportunity to observe the two objects for 45 s before making their choices. Due to expected loss of attention, longer time delays were not used. Because novelty, that is initial performance, is important, each problem was balanced across the two subjects with respect to time delay. When only testing two individuals, we were only able to perform within subject statistical tests. An individual always had the same delay treatment for all six trials within a session. That is, if Igelchen had time-delay on a specific problem, say problem one of the rope task, then Naong did not have the time-delay on that same problem, and vice versa. Because we only tested two individuals, we could only perform statistical tests within individuals.

It was critical to know that the orangutans had the potential to solve all problems and that they always preferred the rewards over the objects themselves and thus were motivated to try and solve the tasks during all trials. Therefore, prior to each session we subjected the orangutans to one exposure of a functional tool and scored whether they were capable of handling it to reach a reward and, preference tests were made where the object presented in that task was offered next to a peanut (the reward used), in a similar two-choice task. All problems were well within the limits of what these orangutans were capable of.

## Results

### Pre-trials and Preference Tests

Preference tests showed that both orangutans always preferred the rewards over the objects, except in the flask task [Naong preferred reward on 67% (*n* = 6) and Igelchen on 50% (*n* = 6) of the trials). We therefore excluded the flask task from further analyses. In pre-trials both orangutans completed 100% of the three remaining tasks (string, rake, and tube) showing that they were capable of using the tools when functional and presented alone.

### Effect of Time on Two Choice Tests

**FIGURE 2:**
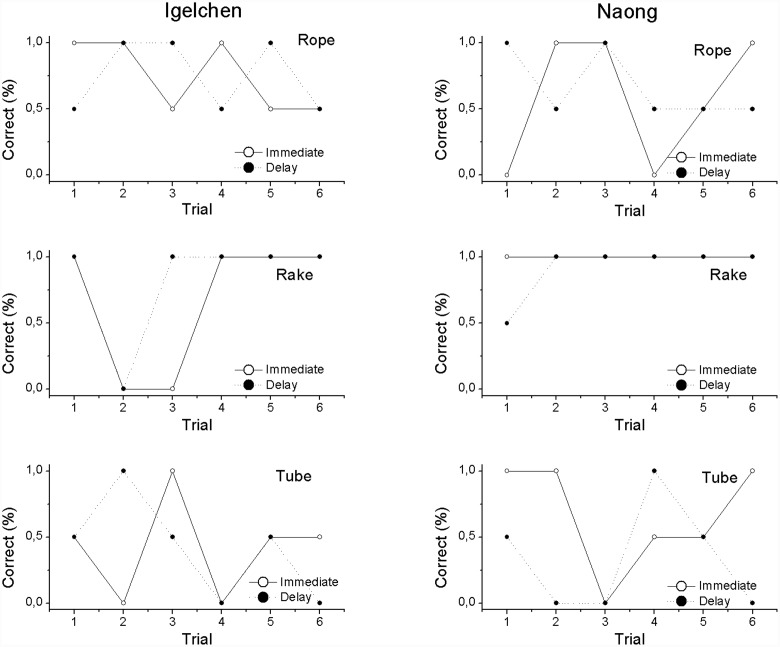
**Results divided across the three different tasks (rope, rake, and tube) for both individuals**. Shown is the difference between choices made in the delay vs. immediate treatments across trials (correct choices were scored as 1 and incorrect choices zero).

**FIGURE 3:**
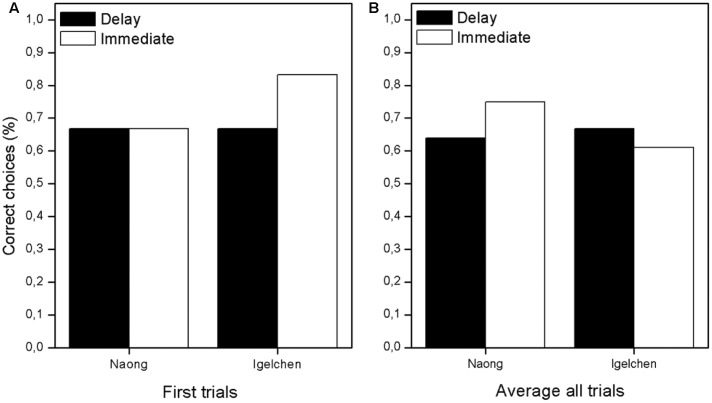
**Overall result showing the success rate in the two treatments. (A)** Average for first trials only in all sessions and **(B)** average based on all six trials for all sessions.

First, we wanted to analyse first attempts, because correct choices were rewarding and therefore affect the remainder of the choices (all trial 1 in **Figure 2**). When comparing, within individual, the trials in the two delay treatments no differences in performance were found (Mann–Whitney *U*-test: Naong: *n* = 12, *U* = 18.0, ns, Igelchen: *n* = 12, *U* = 21.0, ns, **Figure 3**). No differences were found when we tested the average performance for all six attempts within a test session, as described in **Figure 2**. Thus, we found no increase in performance when the orangutans had the opportunity to examine the task visually for a longer period of time (Naong: *n* = 12, *U* = 22.0, ns, Igelchen: *n* = 12, *U* = 20.0, ns), **Figure 3**.

### Side Preference Test

No significant difference was found between choosing objects on the right or the left side for any of the orangutans (Mann–Whitney *U*-test: Naong: *n*1 = 12, *n*2 = 12, *U* = 106.0, ns, Igelchen: *n*1 = 12, *n*2 = 12, *U* = 102.0, ns).

### Results Averaged Over Trials

In general, the orangutans were not very successful in solving these tasks. Regardless of time treatment, we report results averaged over the six trials in **Table 1**. Igelchen performed above 80% in four of 12 tasks and Naong performed at 80% and above in five of 12 tasks. We exclude flask tasks for reasons mentioned above.

**Table 1:** Results in % correct choices for both orangutans in average over the six trials for each respective task.

Task	Rope		Rake		Tube	
	Igelchen	Naong	Igelchen	Naong	Igelchen	Naong
1	50	50	67	80	33	67
2	100	83	67	100	33	17
3	50	50	83	100	50	67
4	100	67	83	100	50	50
Mean	75	63	75	95	42	50

### A Note on Attention

For a prolonged viewing time to be useful the orangutans had to pay attention. Both individuals had extensive experience from choosing between different objects prior to this experiment. We have not had the opportunity to quantify exactly what they were looking at during the experiments, but observations during the experiment tell us that upon withdrawal of the opaque screen their apparent attention was, at all trials, directed toward the tray in front of them. We do not see any reasons to suspect that the results were biased due to a lack of attention during delay trials.

## Discussion

Here, we have described a way whereby reasoning capacities can be identified by studying if more time for decision making improves performance in a two-choice problem solving task. In contrast to problem solving in humans, our results do not indicate that more time allowed for solving a problem increases the performance of orangutans. But, it should be noted that this negative result does not necessarily inform us about mechanisms.

We also found that that these kinds of two-choice problems are difficult, and that orangutans cannot simply look at different options and make decisions to collect rewards at high success rates (**Table 1**). There could be other causes for the negative results than an absence of a capacity for late binding in orangutans, as these tasks could either have been too simple or too difficult. If tasks were too simple, choices would be correct irrespective of time delays, and the distribution of choices would be highly skewed. This did not seem to be a problem as the distribution of the number of correct choices made per session did not deviate from normal (Kolmogorov–Smirnov test, *D* = 0.15, *p* = 0.21). If the tasks were too difficult we would expect the orangutans to choose at random, and this was not the case (**Table 1**).

A similar study has been made on chimpanzees, orangutans and human children. Here, subjects were allowed to observe puzzle boxes at different intervals before opening them (sometimes allowed 24 h or 48 h prior view of boxes vs. immediate presentations). The authors suggested that apes are not capable of mental rehearsal (Dunbar et al., 2005), but unfortunately the design of that study rendered their result uncertain. In chimpanzees, different puzzle boxes were used in different treatments so effects of treatment could not be separated from effects of variation in puzzle boxes. In addition, as the authors wrote “However, the design of the experiment inevitably means that practice effects may confound these results” (p. 327).

There is room for improvement of these kinds of studies. First, by studying attention, and what animals can learn from just observing, would help improving the experimental set-up. A better understanding of what kind of objects attract the attention of different animals would also be helpful. This could make the tasks better suited for the species in question, but at the same time such specific methods might not work as well for many species. In addition, it could be valuable to test whether measurements of performance covaries with measurements of attention. Choosing relevant tasks is also an important issue and here, tests, or inspiration, from the literature would be fruitful (e.g., Mulcahy and Call, 2006; Martin-Ordas et al., 2008; Mulcahy et al., 2013, and references therein). Future studies can also be improved by taking into consideration how animals, such as apes, recognize tools that are functional (e.g., Mulcahy et al., 2013). But, irrespective of the choice of tasks, the key manipulation is to test with and without time delay, as this test can thereby determine if time can improve the performance of animal decision making. And, with respect to time delays we are most interested in longer delays, from many seconds to minutes, to enable comparison with humans that are capable of late binding. The method we have described here has a few advantages. First, methodologically, it is easier to exclude effects of individual exploration, which matters both when comparing individual performance and differences between species. Exploration is well known to matter for problem solving (Benson-Amram and Holekamp, 2012) and it varies enormously between species (Glickman and Sroges, 1966). Second, if animals are capable of late binding, it can be detected unambiguously using this kind of method, thereby overcoming the problems of ontogeny and generalization that has rendered other tests ambiguous (see e.g., Anthony, 1959; Jensen, 2006; Lind et al., 2009; von Bayern et al., 2009; Heyes, 2012).

One could argue that imposing delays upon decisions does not have to reveal what mechanisms drive choice behavior. One factor that could make correct choices difficult is if subjects cannot inhibit previously rewarded responses (see discussions in, e.g., Povinelli, 2000; Girndt et al., 2008). The appearance of inhibition problems can potentially be predicted from learning models that include some decision rule, and if there is knowledge about stored values of behaviors (see e.g., how the decision rule interacts with values of behavior in Enquist et al., 2016). But, the aim of our study was to use a method with the potential of detecting whether a delay could improve decisions. Our method does not permit conclusions about how previous experiences caused the negative finding.

In relation to learning and decision making that goes beyond standard associative learning we would like to highlight another paradigm that has methodological potential, but has been neglected within animal cognition, namely outcome revaluation studies (e.g., Chen and Amsel, 1980; Adams and Dickinson, 1981; Kenward et al., 2009). These studies have, to the best of our knowledge, provided the best examples of reorganization of information in animals. However, these experiments have not yet provided clear evidence for mechanisms underlying reasoning, because one can only see gradually inhibited responses and not the novel, qualitatively different, responses that are to be expected from a mechanism underlying reasoning. But, we believe this paradigm has great potential for studying animals’ capacity for reorganizing information because it provides a methodology that permits clear interpretation of the experimental outcomes. It can also be used to test explicit hypotheses about how information is processed because experiences are well controlled, tests are made under extinction and hidden learning phenomena can be exposed during reacquisition phases.

## Conclusion

Our results did not show that orangutans performed better in two-choice task when allowed more time before decisions were made. However, we have described a method that can prove useful for future research into processes of reasoning in non-human animals.

## Author Contributions

All authors listed, have made substantial, direct and intellectual contribution to the work, and approved it for publication.

## Conflict of Interest Statement

The authors declare that the research was conducted in the absence of any commercial or financial relationships that could be construed as a potential conflict of interest.
